# Mechanical Properties of Al Matrix Composite Enhanced by In Situ Formed SiC, MgAl_2_O_4,_ and MgO via Casting Process

**DOI:** 10.3390/ma14071767

**Published:** 2021-04-02

**Authors:** Yuhong Jiao, Jianfeng Zhu, Xuelin Li, Chunjie Shi, Bo Lu, Fen Wang, Waras Abdul

**Affiliations:** 1 Shaanxi Key Laboratory of Green Preparation and Functionalization for Inorganic Materials, School of Materials Science and Engineering, Shaanxi University of Science & Technology, Xi’an 710021, China; bs1802010@sust.edu.cn (X.L.); 1602002@sust.edu.cn (B.L.); wangf@sust.edu.cn (F.W.); 90023@sust.edu.cn (W.A.); 2 School of Material and Chemical Engineering, Bengbu University, Bengbu 233000, China; scj@bbc.edu.cn

**Keywords:** SiC/MgAl_2_O_4_/MgO/Al–Mg–Si composite, in situ growth, flash pyrolysis of phenolic resin, casting process, mechanical strength

## Abstract

Al matrix composite, reinforced with the in situ synthesized 3C–SiC, MgAl_2_O_4,_ and MgO grains, was produced via the casting process using phenolic resin pyrolysis products in flash mode. The contents and microstructure of the composites’ fracture characteristics were analyzed by X-ray diffraction (XRD) and scanning electron microscopy (SEM). Mechanical properties were tested by universal testing machine. Owing to the strong propulsion formed in turbulent flow in the pyrolysis process, nano-ceramic grains were formed in the resin pyrolysis process and simultaneously were homogeneously scattered in the alloy matrix. Thermodynamic calculation supported that the gas products, as carbon and oxygen sources, had a different chemical activity on in situ growth. In addition, ceramic (3C–SiC, MgAl_2_O_4,_ and MgO) grains have discrepant contents. Resin pyrolysis in the molten alloy decreased oxide slag but increased pores in the alloy matrix. Tensile strength (142.6 ± 3.5 MPa) had no change due to the cooperative action of increased pores and fine grains; the bending and compression strength was increasing under increased contents of ceramic grains; the maximum bending strength was 378.2 MPa in 1.5% resin-added samples; and the maximum compression strength was 299.4 MPa. Lath-shaped Si was the primary effect factor of mechanical properties. The failure mechanism was controlled by transcrystalline rupture mechanism. We explain that the effects of the ceramic grains formed in the hot process at the condition of the resin exist in mold or other accessory materials. Meanwhile, a novel ceramic-reinforced Al matrix was provided. The organic gas was an excellent source of carbon, nitrogen, and oxygen to in situ ceramic grains in Al alloy.

## 1. Introduction

Ceramic-reinforced aluminum matrix composites play a significant role in structural applications as high specific strength, stiffness, and specific modulus [1]. The major barrier to improving the properties of the ceramic-reinforced Al matrix composite is weak wettability and the harmful interfacial reaction on the interface. In particular, SiC/Al matrix composite, as the most successful ceramic-reinforced Al matrix composites struggled with these conundrums in recent decades [2,3]. Efficient approaches, such as the addition of alloying elements [4,5], surface modification [6,7], and novel fabrication processes [8,9] can be used to improve wettability and regulate interfacial reactions. In recent studies, the innovative fabrication methods have focused on the interface which has been promoted a large improvement on the mechanical properties of ceramic-reinforced Al matrix composites; for instance, accumulative roll bonding [10,11], powder metallurgy technique [12,13], liquid metal infiltration [14,15], selective laser melting [16,17], plasma spraying [18,19], in situ growth [20,21], and special casting. All the above routes enhanced wettability or inhibited interfacial reactions, improving the properties to some extent. Nevertheless, the demand for the high plastic deformation of the matrix in roll bonding, the complex pretreatment of surface modification in powder metallurgy and liquid infiltration (or extra compression), surface hardness improved in plasma spray, the absence of suitable carbon source in the in situ process, or the need for complex equipment in special casting, are among the many limitations of these routes which are obvious.

The casting process, the most frequently used process in metal fabrication, provides the simplest and the most convenient preparation of Al matrix composites [22]. However, it still restricts the application and fabrication of ceramic-reinforced Al matrix composite owing to weak wettability and dispersion. Exceptional casting processes, such as stir casting [22], squeeze casting [23], freeze-casting [24], ultrasonic-assistant casting [25] etc., have emerged as outstanding processes on fabricating ceramic-reinforced Al matrix composites. Nevertheless, these processes have not been entirely disposed restrictions resulting in weak wettability and interfacial reactions.

Depending on previous work [21,26,27,28], in situ synthesized SiC/Al matrix composite has enormous potential in solving the ultimate interfacial predicament. Meanwhile, the in situ synthesizing SiC in a range from the nanometer to micrometer scale has good dispersion in the matrix alloy. However, because of the extremely lower solubility in molten Al, a suitable carbon source was hardly found in the in situ growth processes of SiC. The reverse interfacial reaction between SiC and Al occurred at a high Si concentration in the slurry, which provided a novel approach to in situ formed SiC. The constrained condition of the approach was the inadequacy of the thorough reaction, of which the residue of the Al_4_C_3_ decreased the stability and strength of the composites. As a superior carbon source of the in situ growth in SiC, the phenolic resin provided higher reactivity and higher residual carbon during pyrolysis. However, widely diverse products in different pyrolysis modes obscured the mechanism of the in situ ceramic growth.

Herein, 3C–SiC, MgAl_2_O_4_, MgO-reinforced Al matrix composites were fabricated via the casting process. The phenolic resin was used as an oxygen and carbon source for in situ ceramics in flash pyrolysis mode. According to the pyrolysis products of phenolic resin in the slurry, the mechanism of in situ growth was investigated through the thermodynamic calculation of the designed reactions. The proof of the in situ growth of 3C–SiC, MgAl_2_O_4_, and MgO in flash resin pyrolysis was provided. It is a significant reference to explain the ceramic grains formed in the hot process at the condition of the resin existing in the mold or another accessory material. Additionally, a novel ceramic-reinforced Al matrix was provided. The organic gas is an excellent source of carbon, nitrogen, and oxygen to in situ ceramic grains in Al alloy.

## 2. Materials and Methods

### 2.1. Materials

Al5Mg (99.9%, from the Northwest Institute for Nonferrous Metal Research, Xi’an, Shanxi, China) and Si (99.9%, from the Northwest Institute for Nonferrous Metal Research) ingots were put in a crucible (from the Northwest Institute for Nonferrous Metal Research, Xi’an, Shanxi, China) after being weighed. We placed them in a furnace (Hefei KeJing, Hefei, Anhui, China) which we heated to 800 °C at a rate of 8–10 °C/min. Block phenolic resin (PF2130, Xi’an Resin Plant, Xi’an, China) was fastened on the spoon after being weighed.

### 2.2. Casting Process

**Figure 1:**
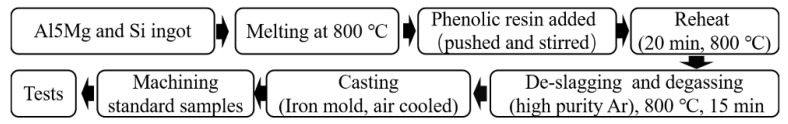
The schedule of 3C–SiC/MgAl_2_O_4_/MgO/Al–Mg–Si fabricated process.

The traditional casting process was used to prepare the 3C–SiC/MgO/MgAl_2_O_4_/Al–Mg–Si composites, as shown in Figure 1. Since the ingots were completely melting, phenolic resin was added by the tailored spoon (iron, like an inverted funnel, Shaanxi key laboratory of green preparation and functionalization for inorganic materials, Xi’an, Shanxi, China), which has kept pushing and stirring. In flash pyrolysis (PF2130, Xi’an Resin Plant, Xi’an, Shanxi, China), violently releasing gaseous products rolled the slurry boiling. Meanwhile, reactions of the in situ growth of 3C–SiC, MgO, and MgAl_2_O_4_ occurred on the interface of the gas and slurry. The stirring process promoted the in situ formed reactions owing to the vast eddy cavities which improved the contact areas of pyrolytic resin in slurry smashed and elongated gaseous pores. The crucible was reheated at 800 °C for 20 min, de-slagging and degassing (high purity Ar) process was finished in the next 20 min at 800 °C. The vibration table (ZD-TW 1-3000HZ, Shanghai Luxuan Instrument Equipment Company, Shanghai, China) was used to reduce the air entrapment. The slurry was filled into an iron mold (Xi’an Feno Oil Gas Technology Co., Ltd, Xi’an, Shanxi, China) which has the dimension of diameter × length = 30 × 350 mm^2^ inner cavity. After demolding, the casting ingots were cooled in the air. In order to obtain mechanical properties, the ingots were cut and machined (SK-50C, Baoji machine Tool Group Co., Ltd., Baoji, Shanxi, China) strictly according to standard samples. All the mechanical processes were under oil cooling agent (FDB7025, Shanghai Baifudun Lubricants oils Co., Ltd, Shanghai, China).

The resin was added as the carbon and oxygen source in the range of 0.3, 0.9, 1.5, and 2.1%, whilst the contents of the matrix alloy listed in Table 1. Bal. is the balance of contents.

### 2.3. Test

**Figure 2:**
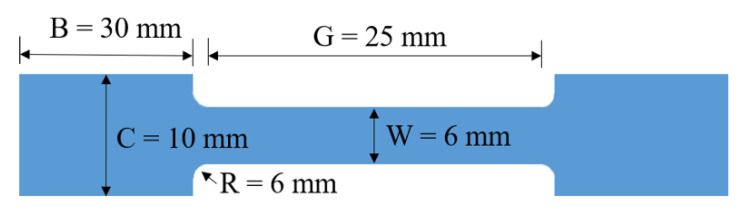
Size details of the samples for tensile test.

To identify the phase constituents, different resin added samples were detected by XRD (Rigaku D/max2200PC, Cu Kα radiation, Tokyo, Japan). The microstructure details of Al and ceramics were characterized by field emission scanning electron microscopy (FESEM, Vision 460, Hillsboro, OR, USA). The strength curves were obtained by the results of the Universal Testing Machine (PT-1036PC, Perfect Internationa Instruments Co., Ltd., Taichung, China) detected; the detail of the sample size was shown in Figure 2. The displacement speed of the load was 0.5 mm/s (maximum load capacity of 10 kN), and each mechanical property was tested three times under identical conditions.

## 3. Results and Discussion

### 3.1. Phase Evolution and Reactions in In Situ Growth Process Based on Flash Pyrolysis Mode

Commonly, phenolic resin has been used as a binder in the sand mold process. The heat in the slurry fills the sand mold, decomposing the phenolic resin in the casting process. Furthermore, phenolic resin has been widely used for composite materials in the aerospace industry, which attracted scholars’ focus on the pyrolysis of phenolic resin. Details of the pyrolysis products on flash mode have been revealed by the designed systematic detectors [29]. Therefore, referenced to the pioneered results, the mechanism of in situ formed ceramic grains was inferred from the phase composition of the composite samples. A plausible explanation established on the designed reaction system whereby the phase composition provided the product of the reaction and the thermodynamic calculated results ensured the forward trend of each reaction, as listed in Table 2. ΔrG is the Gibbs free energy change of the reaction.

**Figure 3:**
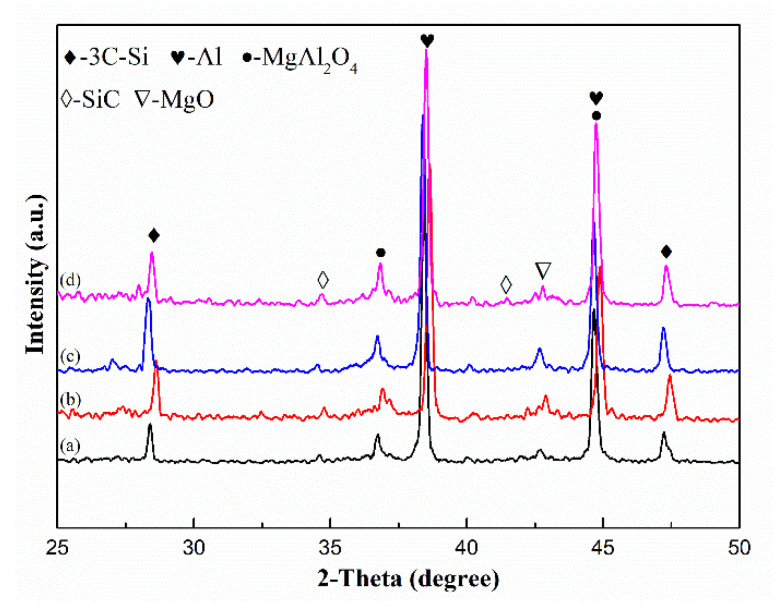
XRD pattern of the composite samples: (a): 0.3%; (b): 0.9%; (c): 1.5%; and (d): 2.1%.

In situ formed MgAl_2_O_4_, MgO, and SiC as reinforcements in the Al–Mg–Si alloy were detected by XRD, as shown in Figure 3. The strongest and second-strongest peaks in XRD curves were the diffraction peaks of the Al (111) plane and Si (111) plane. Weak peaks, identified as the diffraction peaks of MgO, 3C–SiC, and MgAl_2_O_4_, provided proof that in situ growth reactions occurred since the resin addition started. That is, complex mixture gaseous products provided the finite carbon and oxygen source for in situ growth. Meanwhile, a tiny increase in the content of the ceramics depended on a rise in the resin addition. Even if the increase in the resin addition directly extended the contact time, the reaction time would have been restricted to the strong buoyancy, which instantly pumped the gaseous products into the air. That was the primary reason for which ceramics showed weak peaks in XRD results. Furthermore, the finite contact time restricted the continuity of the reactions through the restricted carbon and oxygen source, which restrained the growth and increase in ceramic grains. Additionally, the tiny grains lead to the weakening and broadening peaks in the XRD curve. Moreover, owing to the separated effects, the de-slagging process decreased the ceramic grain content in the slurry. An apparent peak shift (≈0.14° shift to large theta) may have possibly caused the block specimen’s height deviation, as showed in Figure 3b.

It can be included that MgAl_2_O_4_ is the majority phase in the in-situ synthesized ceramics, revealing that MgAl_2_O_4_ formation involved in phenol or phenol derivatives and the Al-Mg alloy is the primary reaction in the process. The direct reasons are its maximum tending of thermodynamic reactions and the major products of gaseous derivative products of phenol and phenol itself. It is preferable to prove that spinel is directly formed in the Al–Mg alloy rather than the pyrolysis obtained MgO hypothesis subsequently reacts with Al (l) and oxygen. It is an undeniable fact that the reaction in which MgO and Al (l) easily produced spinel in an aerobic atmosphere has been proven in other studies. However, one could eloquently argue that direct reactions are more suitable to explain why MgAl_2_O_4_ was the major phase in the condition of inadequate contact time. A similar trend of content between MgO and MgAl_2_O_4_ was revealed by the analogous Gibbs free energy change and the same oxygen source. In situ formed reactions of 3C–SiC hardly discriminated against the carbon source but inferred that benzene and its derivative were due to its higher thermodynamic tendency. A small amount of carbon from the pyrolysis and secondary reactions was considered part of the carbon source, which quickly entered and reacted with the slurry.

The reaction activity and de-slagging process were the additional factors of the ceramic contents. The lower content of 3C–SiC is the minor phase in the in situ synthesis ceramics because benzene and its derivative have slightly lower content and lower tending of thermodynamic reactions. Furthermore, the turbulent gas promoted a complex slurry flow which brought the in situ formed grains to scatter in the slurry homogenously.

### 3.2. The Microstructure Evolution

**Figure 4:**
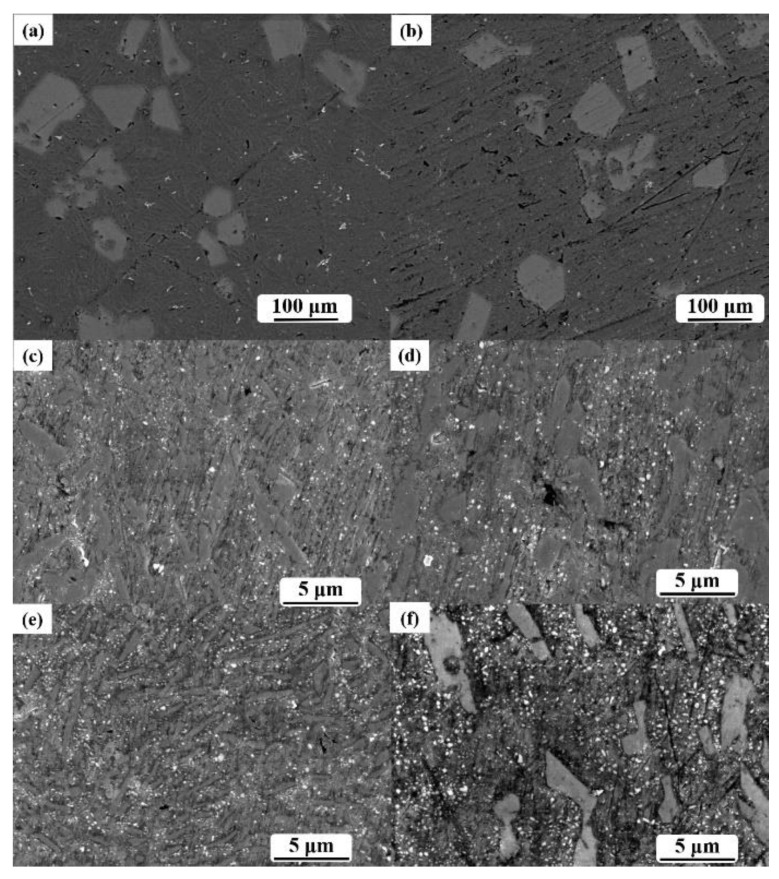
The microstructure of the 3C–SiC/MgAl_2_O_4_/Al composite in different phenolic resin addition: (**a**) blank sample; (**b**,**c**) 0.3% resin added; and (**d**–**f**) 0.9–2.1% resin-added samples.

The increase in resin addition caused the longer reaction time, and further increased the supplied carbon and oxygen source to the in situ reactions. Due to the fact that a change of a minor phase of the XRD results was obscured, the SEM was employed to interpret the changes through the microstructure SEM results, as shown in Figure 4.

The SEM results illuminated the change in ceramic content in the varied addition of resin. The ingots showed provided a compact overview of the microstructure. The shrinkage cavity distribution, slag, and gas entrapment are shown in the blank sample as marked areas in Figure 4a. An apparent discrepancy in anomalous holes and near-sphere holes is shown in Figure 4b. The contents of near-sphere holes formed in the shrinkage in the solidification process were less than the gas entrapment with a larger size and ellipsoid or irregular shape. Increasing contents of ceramic grains raised the viscosity of the slurry while increasing the gas entrapments in the composites.

The size of primary Si was in the range of 20–150 μm, which were the largest grains in the samples. Compared to Figure 4a,b, a demonstrable decrease in slag in ingot was shown after resin addition. It was explained that the large slag particles were brought to the surface of the slurry in stirring. However, gas entrapment was raised owing to Si and ceramic grain which absorbed the released gas on the surface. Due to the stronger bond and a minor amount of gas on the surface of the grain, even though the further process of shaking and degassing were hardly separated, we still observed the tiny gas entrapments. The weak wettability between the ceramic and slurry restricted the abruption of remote gas. The proof is the frequent occurrence of gas entrapment around primary Si. The principal effects of resin addition are increasing gas entrapment while decreasing slag.

With reference to the XRD results, tiny MgAl_2_O_4_, MgO, and 3C–SiC grains were uniformly scattered among the ingots in the size range of 100–300 nm. An obvious increase in the ceramic grains’ contents and size was found in the contrast of Figure 4c–f. The growth explained why the average statistical size of ceramic grains increased in high added resin under the Oswald ripening effect. The smaller the grains that disappeared were, the larger the grains grew. Meanwhile, this is the reason for which the content of ceramic grains showed an indefinite trend in XRD results.

### 3.3. Mechanical Properties

**Figure 5:**
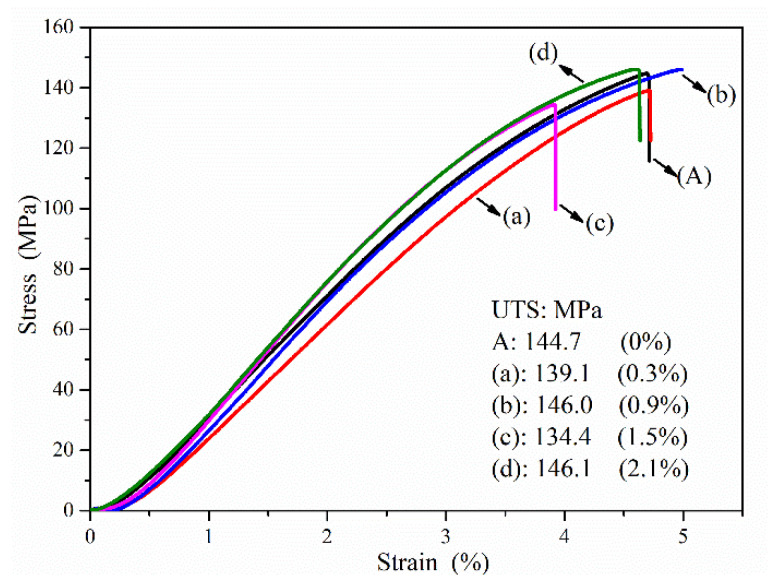
Tensile strength of the strain-stress curve of the samples: (A) is the blank samples; and (a)–(d) are 0.3–2.1% resin-added samples.

The tensile test estimated the strength of the composite at room temperature, as shown in Figure 5. The stress-strain curves showed similar results. The ultimate tensile strength results showed irregular changes under the increase of resin addition. This may be explained by the fact that Si was the primarily affected factor of the tensile strength. The lath-shaped Si grains, as the massive slag, destroyed the homogeneity and continuity of the alloy matrix. Under the tensile stress, the increase in gas entrapment concentrated the area of the original crack. The reinforcements of tensile strength were offset via the increased gas entrapment. The macroscopic fracture showed flat fracture surfaces instead of 45° angles. The bending strength and compression strength estimated the real strength of the composite.

**Figure 6:**
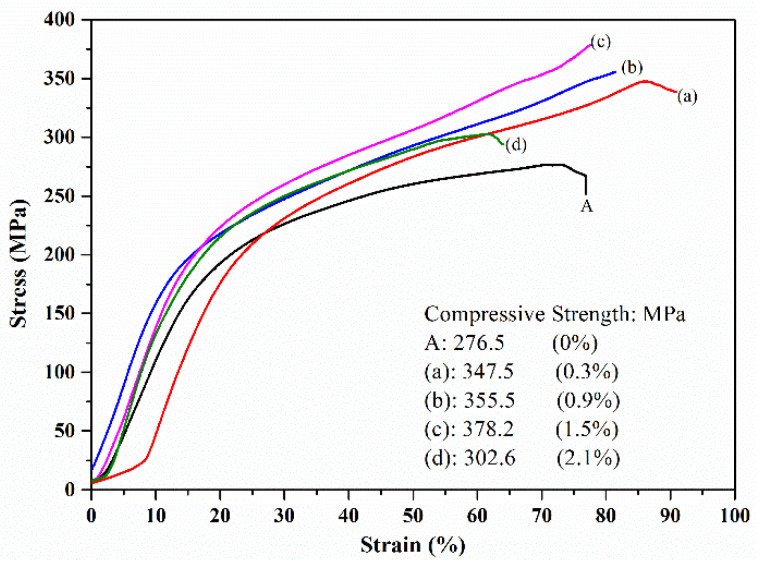
Compression strength of the strain-stress curve of the samples: (A) is the blank samples; and (a)–(d) are the 0.3–2.1% resin-added samples.

**Figure 7:**
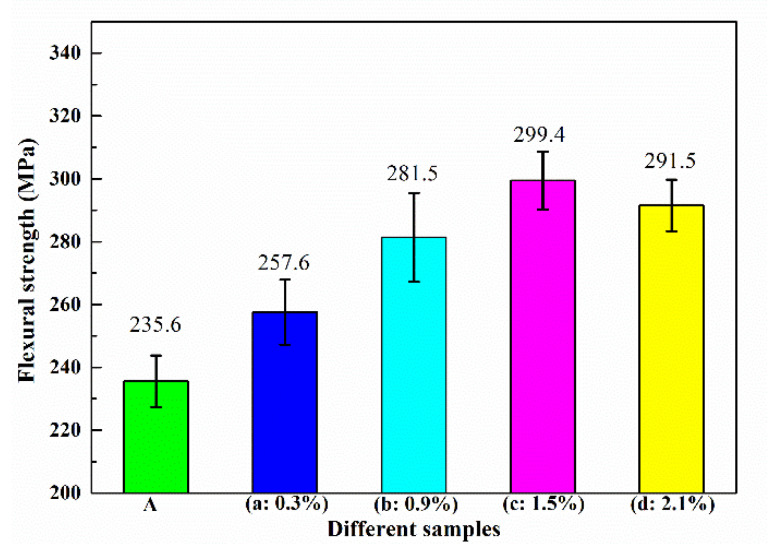
Bending strength of strain-stress curve of the samples: (A) is the blank samples; and (a–d) are the 0.3–2.1% resin-added samples.

The bending strength and compression strength revealed the positive effects of resin pyrolysis, in which the increase in in situ grains growth had an advantage on the strength (Figure 6). Similarly, increased regularity was shown in the compression test (Figure 6), which obviously improved the bending strength and compression strength in the resin-added samples. Compared to blank samples, the bending strength and compression strength of (c) samples (1.5% resin addition) improved to 126.9 and 121.3%, respectively. In the tensile test, the primary adverse effect factor of strength was the lath-shaped Si, but it was a weak influencing factor in the compression test. Based on the same Si content, the bending and compression strength increase was controlled by the increasing in situ growth in ceramic grains. The raised ceramic grains and pores in the matrix alloy provided the supporting structure and compression zone, respectively, as well as restricted the slip movement. This revealed the reinforcement mechanism that the primary effects factor of the bending and compression strength was the increased ceramic grains. Additionally, the difference among the strains in the tensile and compression tests supports the above. Furthermore, since increasing the contents of ceramics grain raised the contents of holes, the increased resin addition decreased the bending and compression strength, as shown in Figure 6 (d: 2.1%) and Figure 7 (d: 2.1%).

Though the tensile strength is lower than that in novel processes [10,30,31,32], the bending strength and compression strength were greatly improved. The discrepancy in compression and tensile strength revealed a conversion in which the plasticity composite showed a small part of brittleness features after the resin addition.

### 3.4. Failure Analysis

The reinforced mechanism of the ceramic grains in the in situ growth process was revealed through the microstructure of a fracture.

**Figure 8:**
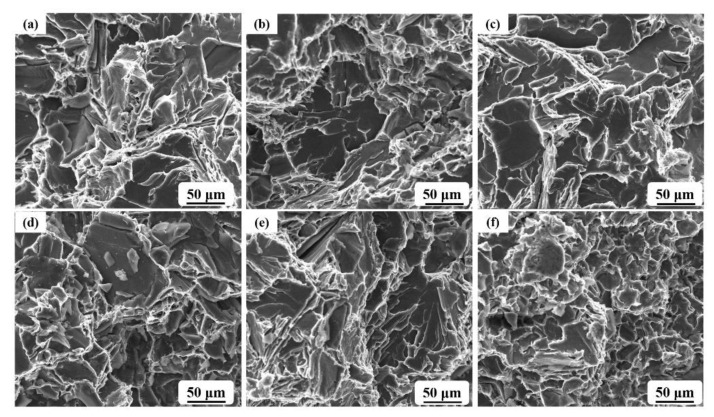
Fracture morphology of different phenolic resin addition samples: (**a**) blank sample; (**b**,**c**) 0.3% resin added; and (**d**–**f**) are 0.9–2.1% resin added samples.

The microstructure of the fractured surface was detected after the tensile test, as shown in Figure 8. It was revealed that large grains of Si controlled the typical brittle rupture of the fracture surface, owing to the size of the large platform which fit the size of the primary Si grains. The tiny grain from the in situ growth process reinforced the composite via the effect of the fine grain. Meanwhile, high hardness grains vastly improved the strength through a deflected microcrack, locked dislocation, and pinned slippage. Large cracks are mainly concentrated around the large platform. The layering steps appeared in the platform; that is, the lath grains of Si fractured in the tensile force. Meanwhile, the cracks extended along with the Si grains’ cleavage facet. The transcrystalline rupture was the primary mechanism resulting from the decreased tensile strength. The compressed force easily impressed the ceramic grains into the matrix in the bending and compression test, and the pinning theory explained the increased strength. Furthermore, the in situ ceramic grains decreased the size of the primary Si crystal, which had an advantage on the reinforcement of composites.

## 4. Conclusions

3C–SiC, MgAl_2_O_4_, and MgO-reinforced Al matrix alloys were synthesized via flash resin pyrolysis in a molten Al alloy. The XRD, SEM, failure microstructure, and strength test revealed the reinforcement mechanism from in situ growth ceramic grains.

1. The nanosized 3C–SiC, MgAl_2_O_4,_ and MgO grains were formed in the Al–Mg–Si matrix alloy by in situ growth process in flash pyrolysis mode. The resin pyrolysis in the molten alloy decreased the oxide slag but increased the pore in the alloy matrix.
2. These ceramic grains scattered in the alloy were homogenous and enhanced the mechanical strength. Except for the tensile strength, the bending and compression strength was increased under the high resin addition.
3. The tensile strength was 142.6 ± 3.5 MPa, and the maximum bending strength and compression strengths were 378.2 MPa and 299 MPa in the 1.5% resin-added samples, which improved to 126.9 and 121.3%, respectively.
4. The lath-shaped Si was the primary effect factor of the mechanical properties. The failure mechanism was controlled by the transcrystalline rupture.

## Figures and Tables

**Table 1:** The contents of the matrix alloy (%).

Elements	Mg	Si	Ti	B	Al
Contents	5	25	0.02–0.06	<0.01	Bal.

**Table 2:** Designed reactions and their Gibbs free energy change (800 °C).

No.	Reactions	ΔrG (kJ)
1	Al + 1/2Mg + 2H_2_O (g) = 1/2MgAl_2_O_4_ + 2H_2_ (g)	−357.05
2	Al + 1/2Mg + 2CO (g) = 1/2MgAl_2_O_4_ + 2C	−320.96
3	Al + 1/2Mg + CO_2_ (g) = 1/2MgAl_2_O_4_ + C	−338.49
4	Al + 1/2Mg + 2C_6_H_6_O (g) = 1/2MgAl_2_O_4_ + 12C + 6H_2_ (g)	−1035.51
5	Al + 1/2Mg + 2C_7_H_8_O (g) = 1/2MgAl_2_O_4_ + 14C + 8H_2_ (g)	−1202.49
6	Al + 1/2Mg + 2C_8_H_10_O (g) = 1/2MgAl_2_O_4_ + 16C + 10H_2_ (g)	−1327.73
7	Mg (l) + C_6_H_6_O (g) = MgO + 6C + 3H_2_ (g)	−605.79
8	Mg (l) + C_7_H_8_O (g) = MgO + 7C + 4H_2_ (g)	−689.28
9	Mg (l) + C_8_H_10_O (g) = MgO + 8C + 5H_2_ (g)	−762.77
10	Si (l) + 1/5C_6_H_6_O (g) =SiC + 3/5H_2_ (g) + 1/5CO (g)	−153.03
11	Si (l) + 1/6C_7_H_8_O (g) = SiC + 2/3H_2_ (g) + 1/6CO (g)	−155.04
12	Si (l) + 1/7C_8_H_10_O (g) = SiC + 5/7H_2_ (g) + 1/7CO (g)	−157.30
13	Si (l) + 1/5C_6_H_6_ (g) = SiC + 3/5H_2_ (g)	−127.15
14	Si (l) + 1/7C_7_H_8_ (g) = SiC + 4/7H_2_ (g)	−129.98
15	Si (l) + 1/8C_8_H_10_ (g) = SiC + 5/8H_2_ (g)	−134.19

## Data Availability

Data sharing not applicable.
